# Risk Attitude in the DuLong Minority Ethnicity of China

**DOI:** 10.3389/fpsyg.2021.596745

**Published:** 2021-02-18

**Authors:** Lili Tan, Siyuan Li, Xiaomin Zhang

**Affiliations:** ^1^School of Public Administration, Yunnan University, Kunming, China; ^2^School of Basic Medicine, Kunming Medical University, Kunming, China

**Keywords:** four-fold risk attitude, prospect theory, DuLong minority ethnicity, choice-based elicitation procedure, non-industrialized small society

## Abstract

Prospect theory predicts a four-fold risk attitude, which means that people are risk seeking for low-probability gain and high-probability loss and risk averse for low-probability loss and high-probability gain because they overweight probability when it is low. The four-fold pattern of risk attitude has been supported by several former studies with mainstream industrialized populations but has never previously been tested in a non-industrialized society. In this work, we examined the robustness of the four-fold risk attitude in the DuLong minority ethnicity in China, which is a small society with only 4,000 members that is isolated from modern civilization. We used simple lotteries for gain and loss with different probabilities to elicit the risk attitude of 37 DuLong villagers. Our results support prospect theory predictions in that DuLong people are risk seeking for low-probability gain and risk averse for low-probability loss. However, although they showed a tendency to decrease their degree of risk seeking (risk aversion) for gain (loss), their risk attitude did not reverse when the probability of the prospect increased to 50%. In summary, our results suggest a right-shifted weighting function in this non-industrialized small society. The deviation might be caused by the particular living situation of the DuLong people, their sensitivity to monetary payoffs, and the elicitation procedure.

## Introduction

Individuals constantly make decisions containing a certain level of risk. However, there is no clear answer to the important question of how to identify a person's risk attitude and whether a person's risk attitudes vary according to the specific situation and time. Expected utility theory (EUT) entails that individuals, when faced with a risky choice, calculate the expected utility of each option, make a comparison, and ultimately choose the option from which they expect the greatest utility. The theory holds that the expected utility is calculated by multiplying the potential outcomes by its probability (von Neumann and Morgenstern, 1944; Bernoulli, 1954). However, in practice, people's choices are often at odds with the prediction of expected utility theory (Allais, 1953; Schoemaker, 1982; Starmer, 2000) that people's risk attitude will be far more complex. Subsequently, Kahneman and Tversky put forward the four-fold pattern risk attitude in their prospect theory (PT) (Kahneman and Tversky, 1979; Tversky and Kahneman, 1992). The four-fold pattern risk attitude suggests that when faced with a risky choice, people will be (1) risk seeking over low-probability gains, (2) risk averse over high-probability gains, (3) risk averse over low-probability loss, and (4) risk seeking over high-probability loss. The authors also claimed that the reason for these phenomena is that people tend to overweight low probability and therefore loss and gain when the probability is extremely low.

To date, the four-fold pattern risk attitude has been verified by several studies. In the study they conducted in 1992, Kahneman and Tversky provided experimental evidence to support this theory through hypothetical lotteries with 25 graduate students (Tversky and Kahneman, 1992). They found that most subjects were risk seeking for gain and risk averse for loss in 1% probability lotteries but reversed their risk attitude in 50% probability lotteries. In second auction experiments in which subjects competed in auctions to buy the right to insure against a potential loss or to assure themselves a gain, Di Mauro and Maffioletti found evidence supporting this theory, and they also found that this risk attitude could extend to ambiguous decision making (Di Mauro and Maffioletti, 2004). Brooks et al. tested the choice behavior of people for pure gain lotteries, pure loss lotteries, or mixed lotteries with both potential gains and losses, and their results supported the predictions of prospect theory (Brooks et al., 2014). A recent study with a total of 4,098 subjects from 19 different regions worldwide confirmed that 94% of the items suggested by PT theory could be successfully replicated, including experiments revealing the four-fold risk attitude (Ruggeri et al., 2020). Several other studies obtained results that were partially consistent with the four-fold hypothesis. Kachelmeier and Shehata observed a risk-seeking attitude for low-probability gain and the tendency to alter risk attitudes from risk seeking to risk neutral and even slightly risk averse, as predicted by PT. However, they did not find strong risk aversion for high-probability gain (Kachelmeier and Shehata, 1992). Holt and Laury tested risk attitude by simple lottery-choice experiments and found that people are risk averse for gain and risk neutral for losses for real gambles but more risk seeking in hypothetical gambles (Holt and Laury, 2002). Harbaugh et al. investigated risk attitude with a group of subjects aged between 5 and 64. The results showed that the risk attitude of children is opposite to the PT predictions, but when people become older, they behave more consistently with the four-fold pattern (Harbaugh et al., 2002). In a later study, it was reported that through a priced-based but not a choice-based elicitation procedure, people's preferences were consistent with the four-fold pattern of risk attitude (Harbaugh et al., 2010). Hertwig et al. also found that people's risk attitude varied according to the elicitation method used in the experiment and that when the probability was directly stated, people made choices that aligned with the four-fold risk attitude; however, when the probability was learned from experience, the pattern reversed (Hertwig et al., 2004, 2019; Hertwig and Erev, 2009). Therefore, they suggested a description-experience gap in risk decision making that might be caused by a number of reasons. For instance, people might rely on small samples when they estimate the probability of choices in experience. It is also possible that the weighting of stated and experienced probability is different (Hertwig and Erev, 2009; Wulff et al., 2018). Overall, the four-fold risk attitude suggested by prospect theory still lacks enough experimental evidence, and the current studies focusing on it have not yet resulted in a consensus.

Moreover, another issue regarding the robustness of the four-fold risk attitude is that, like other psychological and economic experiments, the existing studies rely heavily on college students and faculty. For instance, in their 1992 experiments, Tversky and Kahneman recruited 25 graduate students from Berkeley and Stanford University as their subjects. Halt and Laurys experiments used subjects who were undergraduates, MBA students, or business school faculty (Tversky and Kahneman, 1992). The subjects of the experiments of Brook et al. were graduate and undergraduate students in economics. In Kachelmeier and Shehata's study, the subjects were students from China, Canada, and United States (Kachelmeier and Shehata, 1992). The homogeneity of the subjects may cause more severe bias in the experimental results. A recent study to replicate PT-predicted decisions partly addressed this issue by using a large subject pool from 19 regions that varied in education level and income and encompassed a wider age range. Still, all subjects were from industrialized society. In studies of other psychological or economic issues, it has been suggested that the homogeneity of the subjects might cause an overall bias in the experimental results because the subjects can hardly represent Homo sapiens as a whole. In particular, in most psychological and economic studies, the subjects are western, educated, industrialized, rich, and democratic (WEIRD) populations, which is also true for the studies of the four-fold risk attitude (Henrich et al., 2010a,b). In contrast, people from non-industrialized small societies tend to show different social preferences due to their low market exposure and poor living conditions (Henrich et al., 2001, 2004). For instance, on average, participants from 8 small societies made fewer than 40% of the offer in either the dictator game or the ultimatum game, and the lowest offer was ~25% for both games, which is significantly different from the ~50% offer made by the “WEIRD” population.

Therefore, whether the four-fold risk attitude holds in a non-industrialized small society remains questionable and has not previously been tested. In the present study, we investigated this issue in the DuLong ethnic minority in China. The DuLong valley in Gongshan County of Nujiang Autonomous Prefecture is the main living area of China's DuLong minority ethnicity. It is situated on the border with Myanmar to the west and the neighboring Lhasa city to the north and covers 1,944 square kilometers with ~4,000 residents. At present, the livelihood in DuLong depends mostly on rotational swidden agriculture of several crop types, including maize, millet, and beans, and economic activity is restricted to the exchange of forest products for planting tools with neighboring villages (Gros, 2009; Shen et al., 2010). The DuLong area has long been regarded as an official “primitive society” in China because the extreme mountainous terrain constitutes a natural barrier between it and the economic and cultural development of the outside world, making the DuLong people the most isolated minority ethnicity in China. The first motor road of the DuLong Valley, which is 96 kilometers long, was finished in 1966. This is the most important channel connecting the DuLong people with the wider society. However, due to the complicated geology of the DuLong River area, the frequent mudslides and landslides, and the snow that isolates the mountains for more than half the year, DuLong people rarely leave this area. The education level of the DuLong people is extremely low since their formal modern education started in 2006. Merely 300 DuLong villagers have received a junior school education, and 50 have received a high school education. Furthermore, the majority of DuLong villagers above 30 years old speak only their local language and have never come into contact with modern education. In Table 1, we summarize the features of DuLong society and the living conditions.

**Table 1:** Summary of the dulong society and the environment.

	**Description**
Environment	• In high mountains and deep valleys along the Dulong River • The natural environment is harsh • The average altitude is close to 3,000 meters, the highest altitude is 4,900 meters
Transportation accessibility	• There is only one road open to traffic, and it is only accessible for half a year • Residents rarely leave the area
Language	• Dulong language without words, belong to Sino-Tibetan languages Tibeto-Burman languages • Young people below 30 can understand and speak simple mandarin
Economic base	• Before the 1990s, primitive production activities including hunting and gathering • Since the 1990s, small scale farming has gradually formed, and cash started to be used
Income	• 2,000–3,000 RMB per year, mainly government subsidy • Rarely involved in paid job
Education	• The modern education system started in 2006 • Less than 10% population received junior school level education • Less than 1% population received high school level education
Exposure to great loss	• Higher exposure to great loss, compare to people in mainstream industrial society • High risk of accidental death: fall off a cliff, stung by wild bee, attacked by a beast
Living resources accessibility	• Limited and less reliable living resources

It is worth noting the two major differences between the living conditions of DuLong society and industrialized mainstream society: the DuLong people are often exposed to great losses, and their living resources are unstable. On the one hand, on average, DuLong people experience great losses more frequently. In a survey of 124 elementary and junior students, the incidence of parental death was more than 15%. On the other hand, for DuLong people have long relied heavily on hunting and gathering for survival and obtaining living resources is very unreliable. Although they gradually turned to small-scale industry after the 1990s, the stability of agricultural production remains low due to the limitations of the natural climate conditions and agricultural technology. Whether people of this small and isolated society, with living conditions quite distinct from those of industrialized modern society, develop the same risk attitude as WEIRD subjects is questionable. We speculated that their primitive and highly unstable living situation would influence their risk attitude. Whether this is true and the direction of the alteration are worth investigating. Therefore, in this study, we used a simple lottery choice to test the risk attitude of DuLong people toward gain and loss. We found that at the aggregate level, the subjects are risk seeking for gain and risk averse for loss when the probability is low, and we also found a risk-neutral tendency when the probability is enlarged, but we did not find a reversal of their risk attitude. Therefore, our results partially support the predictors of PT's four-fold pattern of risk attitude.

## Methods

### Ethnic Statement

Each subject provided written informed consent to participate in the experiment. The use of subject payment incentives in the experiment was approved by the Medical Ethnic Committee of Kunming Medical University.

### Subjects

A cohort of 37 DuLong Chinese subjects in the DuLong River of Yunnan Province was recruited in November 2018. The demographics of these subjects are summarized as follows: mean age 23 years old (range: 19–40 years old), with 21 males and 16 females. All subjects had a primary school education. None of the subjects had paid jobs or had ever been involved in paid jobs. Their annual monetary income was between 2,000 and 3,000 RMB, which came mostly from government subsidies. The investigators adhered to the practice in experimental economics of applying monetary incentives to motivate decision making without using deception.

### Experimental Design

The investigators used four sets of simple choice tasks to represent the lottery, according to the previous literature, in the form of questionnaires (Cohen et al., 1987; Tversky and Kahneman, 1992; Holt and Laury, 2002; Zhong et al., 2009). Each task contained 10 pairs of choices with a lottery and a certain outcome. The lottery did not change in the 10 pairs of choices, but from the first to last pair of choice, the certain outcome gradually increased in the pure gain experiments and gradually decreased in the pure loss experiments. Detailed information on the four tasks can be found in Appendix Tables 1–4.

The experiment was conducted in a one-to-one manner. For each subject, the experimenter individually filled out the questionnaire with him or her. Because the language commonly used by the DuLong villagers is the DuLong language, some subjects did not speak Chinese well, and their literacy was very limited. Therefore, to ensure that the subjects could fully understand the content of the questionnaire and the choices to be made in each experiment, we were equipped with a translator proficient in both DuLong and Mandarin to accompany the experiment. In addition, all the experimental instruments were explained to the subjects orally. The following is the content of the instrument of moderate probability pure gain lottery.

*In this scenario, there are 100 cards:50 black cards and 50 red cards. Please randomly select one of them and guess whether it will be a black card or a red card before the draw*.

*Option A: If the card your chose is red, you will get 100 RMB; if it is black, you will get 0 RMB. In other words, you have a 50% probability of getting 100 RMB and a 50% probability of getting 0 RMB*.

*Option B: Ten types of amounts are listed (in ascending order), corresponding to the amount you will definitely get if you choose this item*.

*Decision: For the following 10 rows, please mark your choice with a check mark (*√*) in the last column of each row*.

To ensure that the subjects correctly understood the probability in the experiment, we conducted the experiment by drawing playing cards. Changing the probability was achieved by changing the number of black and red cards in the deck.

Each participant received a participation fee of 20 RMB. In addition, after the experiment was over, the experimenter randomly selected one of the 40 choices in the four experiments and made an incremental payment according to the choices of the subjects. All incentives were paid in cash immediately when the subjects finished the whole experiment.

The risk attitude is decided following former studies (Tversky and Kahneman, 1992; Holt and Laury, 2002). In all of our treatments, the majority of the subjects chose option A at the beginning and then crossed over to option B without ever returning to option A. There were a few cases of all-A or all-B choices. The total number of “risky” A choices is an indicator of the degree of risk seeking. In the 4th pair of each task, the EV (expected value) of the lottery (calculated according to the Von Neumann–Morgenstern utility theorem with risk-neutral assumption) equals the certain outcome.

Therefore, at the aggregate level, a risk-neutral cohort should choose option A in the first three pairs of choices and switch to option B at the 5th pair of choices. The risk-neutral subjects should randomly choose A or B in the 4th pair of choices because the two options are equal to each other. At the individual level, the risk attitude is measured by comparing the EV and the CE of the lotteries for each subject. The risk attitude depends on the switch point of the subject in each experiment. For example, in the MG experiment, if the subject chooses A in the first five pairs of choices and then switches to B at the 6th pair of choices, then her CE for this lottery is calculated by dividing the sum of the certain outcomes of the 5th and 6th pairs of choices. The calculated CE is listed in Appendix Tables 1–4. According to our experimental design, if the subject switches his/her choice from A to B (the relationship between switch point and individual risk attitude is summarized in Table 2):

*Before the 4th trial (included), the individual is risk averse*,

Or if

*After the 5th trial (included), the individual is risk seeking*.

**Table 2:** The risk attitude ate individual level.

**Treatment**	**Choice behavior: Time** **for switch from** **option A to option B**	**Risk attitude**
LG	Before the 4th choice (included) After the 5th choice (included)	Aversion Seeking
LL	Before the 4th choice (included) After the 5th choice (included)	Aversion Seeking
MG	Before the 4th choice (included) After the 5th choice (included)	Aversion Seeking
ML	Before the 4th choice (included) After the 5th choice (included)	Aversion Seeking

## Results

In the present study, we used choices in lotteries with real monetary rewards to elicit risk attitudes in the DuLong minority ethnic group, which is the most isolated and non-industrialized minority ethnicity in China.

### Analysis of Choice Over Gains and Losses

We conducted four sets of experiments: moderate-probability pure gain lottery (MG), low-probability pure gain lottery (LG), moderate-probability pure loss lottery (ML), and low-probability pure loss lottery (LL). Each set of experiments contained 10 pairs of choices with a lottery (option A) and a certain outcome (option B) (detailed in Appendix Tables 1–4).

**Figure 1:**
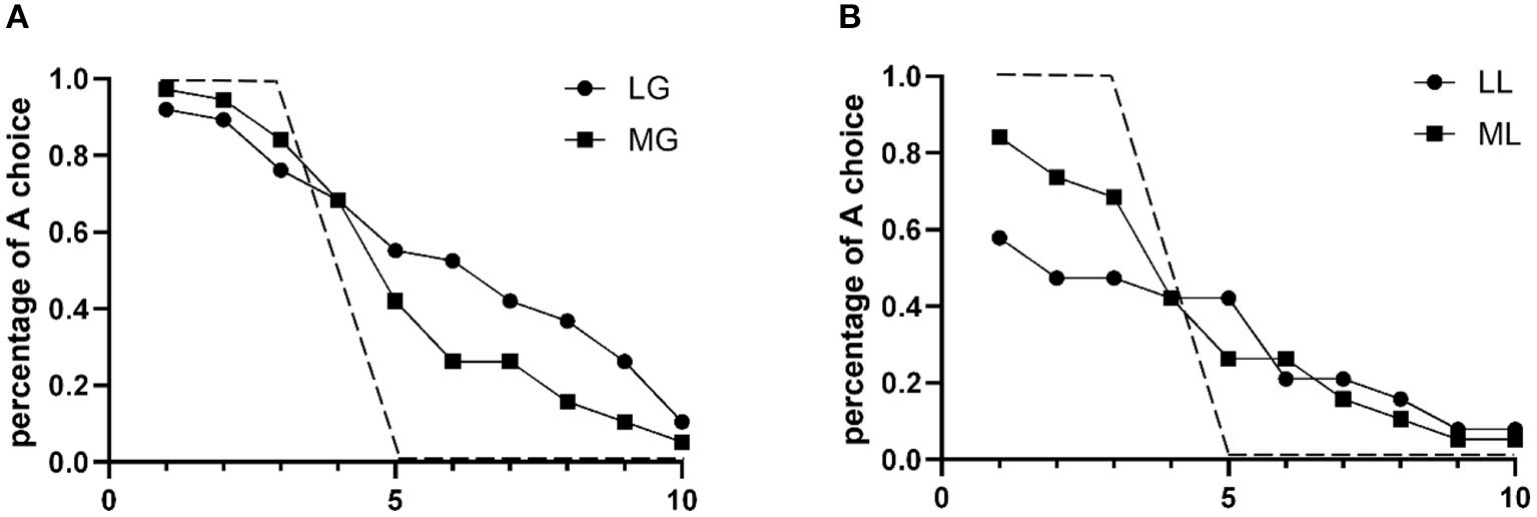
Proportion of risk choices in each decision. **(A)** Data averages for pure gain lotteries. **(B)** Data averages for pure loss lotteries. Solid line with dots represents choices in low-probability lottery; solid line with squares represents choices in moderate-probability lottery. Dashed line represents risk-neutral predictions.

In the pure gain experiments, the expected gain or loss of option A was larger than that of option B in the first three choices, equal to that of option B in the fourth choice, and larger than that of option B in the last six choices. Therefore, as predicted by EUT, a risk-neutral person should choose option A in the first three choices and option B in the 5th−10th choices, as is shown in Figure 1 by the dashed line. We then calculated the percentage of option A, the risk option, in each pair of choices in the two sets of pure gain experiments. As shown in Figure 1A, in the pure gain experiments, the curve of the real choices is to the right of that of the risk-neutral prediction. In other words, in the 5th−10th choices, when the EV of the lottery was smaller than that of the certain outcome, a large number of subjects still chose the risky choice, indicating an overall risk-seeking attitude. Furthermore, we note that the LG and MG curves are not significantly different in the 1st−4th choices, while in the 5th−10th choices, the LG curve is higher than the MG curve, indicating that the subjects were more risk seeking when facing low-probability gain lotteries.

In the pure loss experiments, the expected loss of the lottery in the 1st−3rd choices is smaller than the certain loss. Therefore, risk-neutral subjects should also choose option A in the first 3 choices, and with the gradual decrease in the sure loss, risk-neutral subjects should switch to option B in the 5th−10th pairs of choices. The risk-neutral choice is represented as a dashed line in Figure 1B. Different from the risk-neutral behavior, in our experiment, a large number of subjects chose option B in the first three choices, even though in these three pairs of choices, the certain loss exceeded the expected loss in the lotteries. This result suggests that the DuLong villagers are overall risk averse in pure loss lotteries. With the gradual decrease in certain loss, the percentage of risky choices decreased. This decreasing trend was more significant in the ML experiment, represented as a more sloped curve, which makes it more similar to the risk-neutral curve. In the 1st–3rd choices, the LL curve is lower than the ML curve, which means that more subjects chose B in the LL experiment than in the ML experiment. This result indicates that our subjects showed stronger risk aversion when the probability of the lottery was low.

In addition, the results showed that when the certain loss was already smaller than the gamble, some subjects preferred to bet. It seems that some of our subjects possessed a risk-seeking attitude in loss gambles. Another explanation is that this behavior is a result of loss aversion because the amount of money they will certainly lose when they choose option B was too large. If the subject was motivated by aversion to a certain loss, this effect should be reduced in the LL experiment because the certain loss is much smaller in this experiment than in the ML experiment. However, in the 5th choice, more subjects chose A in the LL experiment, while in the 6th–10th choices, there was no difference between the two experiments, suggesting that a risk-seeking attitude might be the explanation for this phenomenon.

Next, to further characterize the overall risk attitude of the DuLong villagers, we calculated the average number of risky choices in the four treatments. In our experimental design, the EV of the lottery was equal to the certain outcome in the 4th pair of choices, and four risky choices represented the neutral risk attitude of the group as a whole. Thus, the distance to four could represent the level of risk seeking (higher than four) or risk aversion (lower than four). As shown in Table 3, among the 10 pairs of choices in each experiment, the average number of risky choices in the MG experiment is 4.6, compared to 5.5 in the LG experiment, which confirms that our subjects were risk seeking in the pure gain lottery, and the tendency of risk seeking attenuated when the probability increased. In contrast, in the ML experiments, the average number of risky choices was 3.5, while it was 3.1 in the LL experiments, which means that the subjects exhibited a higher level of risk aversion in low-probability pure loss lotteries, although in general, they were risk averse to both loss lotteries.

**Table 3:** Average number of risk choice.

**Treatment**	**Number of subject**	**Number of risk choice**
MG	37	4.6
LG	37	5.5
ML	37	3.5
LL	37	3.1

In summary, these results indicate that the subjects showed different risk attitudes in pure gain and pure loss experiments. In particular, they had an overall risk-seeking attitude in pure gain lotteries and an overall risk-averse attitude in pure loss lotteries. This result violates the EUT prediction that people are always risk averse but is consistent with the PT prediction that when the probability is low, people are risk seeking in the gain domain and risk averse in the loss domain. As explained in prospect theory, this indicates an overweighting of the low probability, which magnifies the utility of the lottery. However, PT predicts a risk-averse attitude in gain and a risk-seeking attitude in loss when the probability is larger than 50%. Several subsequent studies estimated that the weighting function crosses at ~40% (Tversky and Fox, 1995; Prelec, 1998; Gonzalez and Wu, 1999). In general, we found that the DuLong villagers did not change their risk attitude in the ML and MG experiments when the probability increased to 50%. However, we did see a trend of risk attitude change from highly risk seeking (gain) or risk- averse (loss) toward more risk-neutral.

### Individual Behavior in Gambles of Low and Moderate Probability Gains and Losses

**Figure 2:**
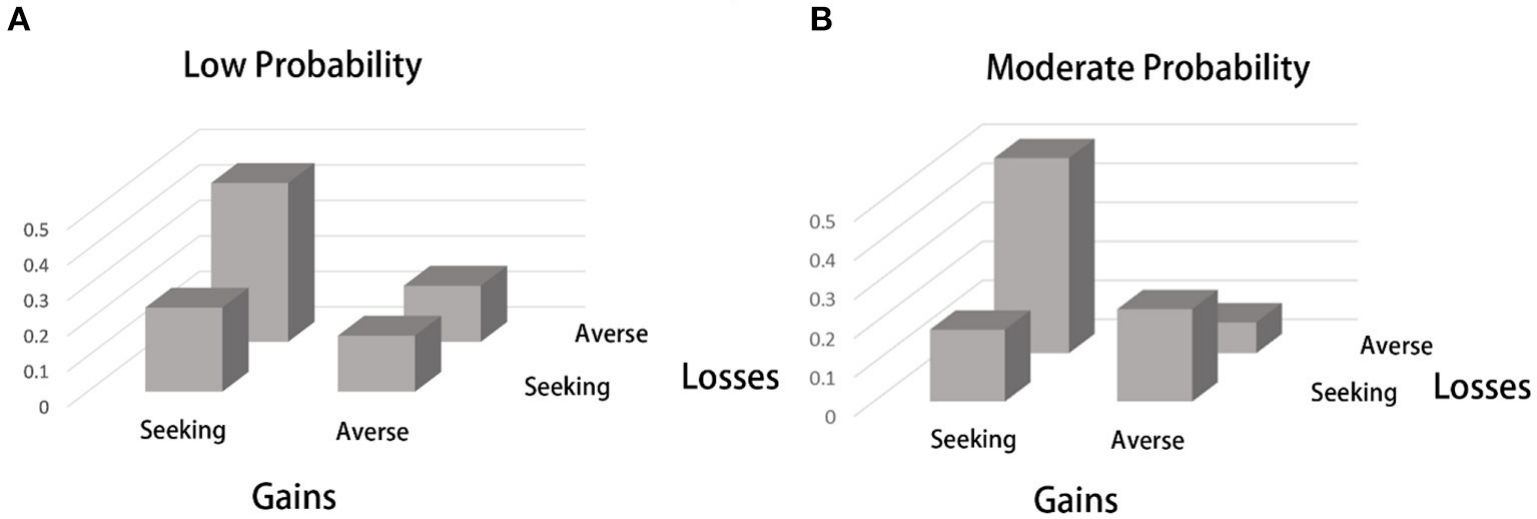
Individual preferences at low- and moderate-probability prospects. **(A)** Numbers of individuals with different risk attitudes in gain and loss lotteries with moderate probabilities. **(B)** Numbers of individuals with different risk attitudes in gain and loss lotteries with low probabilities.

The previous analysis shows that our subjects had a risk-seeking attitude in pure gain experiments and a risk-averse attitude in pure loss experiments, which was more salient in low-probability gambles. However, these experiments were partly inconsistent with the four-fold risk attitude, which is represented as follows: (1) prospect theory predicted a risk-averse attitude in the MG experiment, which was risk seeking in the present experiment, and (2) prospect theory predicted a risk-seeking attitude in the ML experiment, which was risk averse in the present experiment. Therefore, to clarify whether each subject behaved in line with prospect theory, we further analyzed the risk attitude of these subjects at the individual level. We calculated the number of subjects with the four types of attitude in both moderate- and low-probability gambles. As shown in Figure 2A, Table 4, for low-probability lotteries, 17 out of 37 subjects (45.9%), who made up the largest subgroup, were risk seeking for gain and risk averse for loss, which is consistent with the prediction of prospect theory. In moderate-probability lotteries (Figure 2B, Table 5), 18 subjects (48.6%), who made up the largest subgroup, were risk seeking for gain and risk averse for loss, which is contrary to the PT prediction. Meanwhile, nine subjects (24.3%) were risk averse for gain and risk seeking for loss, which is consistent with PT prediction. Since there were two possible risk attitudes for four prospects, there were 16 possible combinations. We found that 11 subjects (29.7%) were risk seeking for gain and risk averse for loss, regardless of the probability of the prospect, and at most three subjects chose any of the other combinations of risk attitudes. Therefore, at the individual level, most subjects behaved consistently with the risk attitude at the aggregated level. It is also worth noting that for moderate probability experiments, about one quarter of the subjects behave consistently to PT prediction.

**Table 4:** Number of people with different risk attitude for low probability experiments.

**Risk attitude**	**Number of people**	**Proportion**
Risk seeking in gains and Risk aversion in loss	17	45.9%
Risk aversion in gains and Risk seeking in loss	6	16.2%
Risk seeking in gains and Risk seeking in loss	8	21.6%
Risk aversion in gains and Risk aversion in loss	6	16.2%

**Table 5:** Number of people with different risk attitude for moderate probability experiments.

**Risk attitude**	**Number of people**	**Proportion**
Risk seeking in gain and Risk aversion in loss	18	48.6%
Risk aversion in gain and Risk seeking in loss	9	24.3%
Risk seeking in gain and Risk seeking in loss	7	18.9%
Risk aversion in gain and Risk aversion in loss	3	8.1%

## Discussion

In the present study, we conducted a series of experiments to verify the four-fold risk attitude in the very closed DuLong ethnic minority group in China. This ethnic minority is located in a mountainous area isolated from urban areas, where small-scale farming that started in the 1990s is the main type of production for the local population, and commercial activities are nearly limited to the mutual exchange of goods with other ethnic minorities in the vicinity. The DuLong residents are low in education, with the majority having received only a primary level of education or below. Ten percent of the population received a junior school education, and <1% received a high school education. For most autumn and winter months, DuLong residents cannot leave their area because the continuous snow seals the only way out, which is a road built in 1966. However, during the rest of the year, even though route is available, they rarely leave the valley. Hence, they have little exposure to modern life and the market economy. In other words, in contrast to the subjects used in previous experiments testing the PT four-fold risk attitude, DuLong is a non-industrialized, small society in China with distinct living condition, few exposures to market, and low education level.

Small societies have been shown to possess distinct social preferences compared to the usual subjects of economic and psychological experiments, who are western, educated, industrialized, rich and democratic people (Henrich et al., 2001, 2004, 2010a,b). In particular, they offer less in the dictator game and ultimatum game, which indicates that they might be less sensitive to fairness (Henrich et al., 2010b) This is attributed mainly to their rare experience with the market economy or industrialized modern society (Gurven, 2004; Tracer, 2004). It is also attributed to their particular social norms, which are distinct from those of modern society, where fairness and cooperation are highly valued (Henrich et al., 2004; Camerer and Fehr, 2005). Whether small societies also possess distinct risk attitudes and whether their risk attitudes comply with the four-fold risk attitude are still unanswered questions. In this work, we examined the robustness of the four-fold risk attitude of a small society, the DuLong people of China, with simple lotteries. The results of this experiment enrich the research in this field and are helpful in exploring the evolutionary conservation of this theory.

Our results indicate that the DuLong cohort possesses different risk attitudes in the gain domain and loss domain and that they are risk seeking in the gain domain and risk averse in the loss domain regardless of the probability. However, we also found that they are less risk seeking (risk averse) in the gain prospect (loss prospect) when the probability of the lottery is 50% than when the probability is quite low, indicating a trend of being more risk neutral when probability increases. Individually, 17 out of 37 subjects were risk seeking in gain and risk averse in loss for low-probability prospects, and 11 of them showed consistent risk attitudes in both low-probability and moderate-probability lotteries, which is the same as the risk attitude of the cohort at the aggregate level. Therefore, the overall risk preference of our subjects as a whole supports the PT prediction in low-probability lotteries but not in moderate-probability lotteries. In general, we did not see a great violation of the PT predictions, only that our subjects, although they showed a tendency to become more risk neutral, did not reverse their risk attitude for over 40% probability, as predicted by PT theory and other previous studies.

PT theory holds that the reason for risk seeking (risk aversion) in low-probability gain (loss) is because the subjects tend to overweight the probability when it is low. Therefore, our results seem to suggest that the DuLong people still overweight the probability when it is relatively large. In other words, the weighting function of the DuLong people is right shifted, so they have a stronger tendency to overweight the probability than PT predicts. This finding is supported by the data that the percentage of risky choice is higher in LG experiment than in MG experiment, while is lower in LL experiment than in ML experiment, meaning that they still applied a PT weighting function only that the cross over point of their risk attitude is delayed.

There are several potential explanations for this deviation from PT predictions and other previous studies. First, the living conditions of DuLong as a non-industrialized small society are quite different from those of mainstream industrial society, especially the chance of confronting great loss and accessibility to living resources. The DuLong people experience great loss more often. A questionnaire survey involving 124 elementary and junior school students organized by one of our authors revealed that 18% of the students had lost their fathers, and more than 15% had lost their mothers. The common cause of death included but was not limited to falling from cliffs or being attacked by wild beasts. A primary school student told us that his father had gone up the mountain to gather fruit and had not returned; he was later found to have been stung to death by wild bees. Furthermore, the DuLong people's living environment is very harsh, and their production methods are primitive, which cause serious bodily injury or even death more frequently than in mainstream industrial society. In mainstream society, the incidence of such great loss is extremely low. It has been proven that people are more risk averse to loss and more risk seeking to gain after disasters because they are more likely to overestimate the probability of rare events (Li et al., 2011). Therefore, we believe that the high exposure to great loss might explain why the DuLong people more significantly overweighted the probability. Moreover, studies of animals have found that when they are in a situation of unstable living resources, they become more risk seeking to gain (Caraco et al., 1980; Moore and Simm, 1985). As mentioned earlier, before the 1990s, the DuLong people still depended mainly on hunting and picking to obtain food and basic living materials. Later, although they gradually turned to small-scale agriculture, the efficiency and stability of agricultural production were low due to the limitations of the natural climate conditions and agricultural technology. Therefore, it is more difficult for the DuLong people to obtain living resources than for those in mainstream industrial society, and the stability of obtaining living resources is much worse. During our visit, most of the DuLong people mentioned strong negative experiences of starvation and freezing. Unstable living resources make them more risk seeking to gain than people in mainstream industrialized society. It is worth noting that the greatly reduced probability of encountering a major loss event and sufficient and stable access to basic living resources are important characteristics and signs of industrialized society. Hence, these two features of the DuLong people might contribute greatly to forming their risk attitude.

In addition, the incentive effect might also play a role. On the one hand, the DuLong people started to use cash extremely late. Before the 1990s, their commercial activity was restricted to barter. After that, although cash was started to be used, they had few chances of purchasing thing by cash. Hence, they might be more (or less) sensitive to monetary payoffs. On the other hand, their annual income is quite low, usually <3,000 RMB and comes mainly from government subsidies; therefore, the potential gain and loss in moderate-probability lotteries was more salient to them than we expected. Although not predicted in PT theory, Holt and Laury did find an incentive effect in risky decisions in that when the payoff increased significantly, risk aversion increased sharply (Holt and Laury, 2002). Kachelmeier and Shehata also found that enlarging the payoff significantly altered the elicited risk attitude. Interestingly, similar to our results, they also observed a decline in the degree of risk seeking when the probability of winning increased and did not see a complete reversal (Kachelmeier and Shehata, 1992). Therefore, if the value of certain loss in the ML experiment ranges from 32 to 25.6 RMB, which is already regarded as a large amount of money by the DuLong people, then their risk attitude would possibly be influenced.

Finally, the elicitation method may also cause the deviation. It has been proven that people's revealed risk preferences could change when elicited in different ways. For instance, Harbaugh et al. found that price-based and choice-based elicitation induced different risk attitudes (Harbaugh et al., 2010). In our experiment, we used only choice-based elicitation, and whether price-based elicitation would generate different results is worth exploring. Moreover, Hertwig et al. found that when the probability was expressed in different ways (description or experience), people showed reversed risk attitudes (Hertwig et al., 2004; Hertwig and Erev, 2009). In our experiment, the probability was directly expressed to the subjects in the form of a poker game, and it is reasonable to believe that DuLong people's risk attitude might differ if the probability were learned by the subjects through some prior sampling experiments. However, although the probability was directly expressed in our experiments, we did notice some daily behavior of the DuLong people that fitted their elicited risk attitudes (some of the authors had lived in the DuLong village for 1 year). For instance, through daily communication, we found that the DuLong people are very sensitive to and afraid of loss of property or illness. In recent years, the government has gradually introduced medical insurance. The DuLong people have a particularly strong willingness to participate in insurance (the insurance fee is RMB 100, about 1/3 of their monthly income), and the participation rate is relatively high (100% among school students). This shows that the DuLong people have more prominent risk aversion in loss. Furthermore, although there is no formal lottery station in the DuLong village, local lottery games where small expenditures may be exchanged for low probability and large returns are quite common in the area, which is consistent with the people's risk-seeking attitude in low-probability gain prospects. In general, our experimental results are roughly consistent with life observations of the DuLong people.

The limitations of the present study include the following: (1) The number of subjects was relatively limited. Since the DuLong ethnic group lacks contact with modern civilization, most villagers can speak only the DuLong language and not Mandarin, and they do not understand basic mathematical principles. Therefore, it is difficult to implement large-scale experiments. However, since the DuLong ethnicity has only 4,000 people, our sample accounts for ~1% of the total population and should therefore be able to roughly reflect the overall characteristics of the DuLong people. (2) In our experimental design, we tested DuLong people's risk attitude only for low and moderate probability lotteries. The limited probability setting does not allow us to estimate the probability weighting function. In future research, we will measure the DuLong people's risk attitude with varying probability in order to estimate their probability weighting function. (3) We used only a choice-based simple lottery in which probability was directly expressed to test the subjects' four-fold risk attitude. Other elicitation methods, as well as other ways of providing probability information, should be tried to confirm the robustness of the results.

## Data Availability Statement

The original contributions generated for the study are included in the article/Supplementary Materials, further inquiries can be directed to the corresponding author/s.

## Ethics Statement

The studies involving human participants were reviewed and approved by Medical Ethics Committee, Kunming Medical University. The patients/participants provided their written informed consent to participate in this study.

## Author Contributions

XZ: conceptualization, writing—review and editing. LT and SL: experiment performing. LT and XZ: data analysis, writing—original draft preparation, and funding acquisition. All authors contributed to the article and approved the submitted version.

## Conflict of Interest

The authors declare that the research was conducted in the absence of any commercial or financial relationships that could be construed as a potential conflict of interest.
