# Supplementary material for: Risk Attitude in the DuLong Minority Ethnicity of China
Source: Front Psychol. 2021 Feb 18;12:596745. doi: 10.3389/fpsyg.2021.596745 (PMC7929998; doi:10.3389/fpsyg.2021.596745)
Supplement: Supplementary file 1 [file Data_Sheet_1.PDF]

# *Appendix*

For an individual in each experiment, he/she will perform 10 choices between a lottery and a sure outcome. If he/she switch his/her choice from A to B

Before the 4th trial (included), the individual is Risk Aversion,

Or if

After the 5th trial (included), the individual is Risk Seeking

The details of the 10 paired choices of each experiment are illustrated in following Tables:

**Table 1. The ten paired lottery-choice decision of low probability pure gain experiment**

| Trial | Option A (RMB) | Option B (RMB) | Expected Value of Gain in Option A (RMB) | Certainty Equivalent (If switch from A to B) (RMB) | Risk Attitude |
|---|---|---|---|---|---|
| 1 | 1% of getting 800; 99% getting 0 | sure gain of 2 | 8 | | |
| 2 | 1% of getting 800; 99% getting 0 | sure gain of 4 | 8 | 3=(2+4)/2 | Aversion (CE < EV) |
| 3 | 1% of getting 800; 99% getting 0 | sure gain of 7.2 | 8 | 5.6=(4+7.2)/2 | Aversion (CE < EV) |
| 4 | 1% of getting 800; 99% getting 0 | sure gain of 8 | 8 | 7.6=(7.2+8)/2 | Aversion (CE < EV) |
| 5 | 1% of getting 800; 99% getting 00 | sure gain of 8.8 | 8 | 8.4=(8+8.8)/2 | Seeking (CE > EV) |
| 6 | 1% of getting 800; 99% getting 0 | sure gain of 12 | 8 | 10.4=(8.8+12)/2 | Seeking (CE > EV) |
| 7 | 1% of getting 800; 99% getting 0 | sure gain of 16 | 8 | 14=(12+16)/2 | Seeking (CE > EV) |
| 8 | 1% of getting 800; 99% getting 0 | sure gain of 22 | 8 | 19=(16+22)/2 | Seeking (CE > EV) |
| 9 | 1% of getting 800; 99% getting 0 0 | sure gain of 28 | 8 | 25=(22+28)/2 | Seeking (CE > EV) |
| 10 | 1% of getting 800; 99% getting 0 | sure gain of 36 | 8 | 32=(28+36)/2 | Seeking (CE > EV) |

**Table 2. The ten paired lottery-choice decision of low probability pure loss experiment**

| Trial | Option A (RMB) | Option B (RMB) | Expected Value of Loss in Option A (RMB) | Certainty Equivalent (If switch from A to B) (RMB) | Risk Attitude |
|---|---|---|---|---|---|
| 1 | 2% of losing 120; 98% of losing 0 | sure loss of 8 | 2.4 | | |
| 2 | 2% of losing 120; 98% of losing 0 | sure loss of 5 | 2.4 | 6.5=(8+5)/2 | Aversion (CE > EV) |
| 3 | 2% of losing 120; 98% of losing 0 | sure loss of 3.2 | 2.4 | 4.1=(5+3.2)/2 | Aversion (CE > EV) |
| 4 | 2% of losing 120; 98% of losing 0 | sure loss of 2.4 | 2.4 | 2.8=(3.2+2.4)/2 | Aversion (CE > EV) |
| 5 | 2% of losing 120; 98% of losing 0ss0 | sure loss of 1.6 | 2.4 | 2=(2.4+1.6)/2 | Seeking (CE < EV) |
| 6 | 2% of losing 120; 98% of losing 0 | sure loss of 1.2 | 2.4 | 1.4=(1.6+1.2)/2 | Seeking (CE < EV) |
| 7 | 2% of losing 120; 98% of losing 0 | sure loss of 1 | 2.4 | 1.1=(1.2+1)/2 | Seeking (CE < EV) |
| 8 | 2% of losing 120; 98% of losing 0 | sure loss of 0.8 | 2.4 | 0.9=(1+0.8)/2 | Seeking (CE < EV)) |
| 9 | 2% of losing 120; 98% of losing 0 | sure loss of 0.6 | 2.4 | 0.7=(0.8+0.6)/2 | Seeking (CE < EV) |
| 10 | 2% of losing 120; 98% of losing 0 | sure loss of 0.4 | 2.4 | 0.5=(0.6+0.4)/2 | Seeking (CE < EV) |

**Table 3. The ten paired lottery-choice decision of moderate probability pure gain experiment**

| Trial | Option A (RMB) | Option B (RMB) | Expected Value of Gain in Option A (RMB) | Certainty Equivalent (If switch from A to B) (RMB) | Risk Attitude |
|---|---|---|---|---|---|
| 1 | 50% of getting 100;<br>50% of getting 0 | sure gain of 30 | 50 | | |
| 2 | 50% of getting 100;<br>50% of getting 0 | sure gain of 40 | 50 | 35=(30+40)/2 | Aversion<br>(CE < EV) |
| 3 | 50% of getting 100;<br>50% of getting 0 | sure gain of 45 | 50 | 42.5=(40+45)/2 | Aversion<br>(CE < EV) |
| 4 | 50% of getting 100;<br>50% of getting 0 | sure gain of 50 | 50 | 47.5=(45+50)/2 | Aversion<br>(CE < EV) |
| 5 | 50% of getting 100;<br>50% of getting 0 | sure gain of 55 | 50 | 52.5=(50+55)/2 | Seeking<br>(CE > EV) |
| 6 | 50% of getting 100;<br>50% of getting 0 | sure gain of 60 | 50 | 57.5=(55+60)/2 | Seeking<br>(CE > EV) |
| 7 | 50% of getting 100;<br>50% of getting 0 | sure gain of 70 | 50 | 65=(60+70)/2 | Seeking<br>(CE > EV) |
| 8 | 50% of getting 100;<br>50% of getting 0 | sure gain of 80 | 50 | 75=(70+80)/2 | Seeking<br>(CE > EV) |
| 9 | 50% of getting 100;<br>50% of getting 0 | sure gain of 90 | 50 | 85=(80+90)/2 | Seeking<br>(CE > EV) |
| 10 | 50% of getting 100;<br>50% of getting 0 | sure gain of 100 | 50 | 95=(90+100)/2 | Seeking<br>(CE > EV) |

**Table 4. The ten paired lottery-choice decision of moderate probability pure loss experiment**

| Trial | Option A(RMB) | Option B(RMB) | Expected Value of Loss in Option A(RMB) | Certainty Equivalent (If switch from A to B) (RMB) | Risk Attitude |
|---|---|---|---|---|---|
| 1 | 50% of losing 60;<br>50% of losing 0 | sure loss of 32 | 30 | | |
| 2 | 50% of losing 60;<br>50% of losing 0 | sure loss of 31.2 | 30 | 31.6=(32+31.2)/2 | Aversion<br>(CE > EV) |
| 3 | 50% of losing 60;<br>50% of losing 0 | sure loss of 30.4 | 30 | 30.8=(31.2+30.4)/2 | Aversion<br>(CE > EV) |
| 4 | 50% of losing 60;<br>50% of losing 0 | sure loss of 30 | 30 | 30.2=(30.4+30)/2 | Aversion<br>(CE > EV) |
| 5 | 50% of losing 60;<br>50% of losing 0 | sure loss of 29.6 | 30 | 29.8=(30+29.6)/2 | Seeking<br>(CE < EV) |
| 6 | 50% of losing 60;<br>50% of losing 0 | sure loss of 28.8 | 30 | 29.2=(29.6+28.8)/2 | Seeking<br>(CE < EV) |
| 7 | 50% of losing 60;<br>50% of losing 0 | sure loss of 28 | 30 | 28.4=(28.8+28)/2 | Seeking<br>(CE < EV) |
| 8 | 50% of losing 60;<br>50% of losing 0 | sure loss of 27.2 | 30 | 27.6=(28+27.2)/2 | Seeking<br>(CE < EV)) |
| 9 | 50% of losing 60;<br>50% of losing 0 | sure loss of 26.4 | 30 | 26.8=(27.2+26.4)/2 | Seeking<br>(CE < EV) |
| 10 | 50% of losing 60;<br>50% of losing 0 | sure loss of 25.6 | 30 | 26=(26.4+25.6)/2 | Seeking<br>(CE < EV) |
